# Management and outcomes of heart failure patients with CKD: experience from an inter‐disciplinary clinic

**DOI:** 10.1002/ehf2.12796

**Published:** 2020-07-11

**Authors:** Mai Nguyen, Samir Rumjaun, Racquel Lowe‐Jones, Irina Chis Ster, Giuseppe Rosano, Lisa Anderson, Debasish Banerjee

**Affiliations:** ^1^ Renal and Transplantation Unit St George's University Hospital NHS Foundation Trust London UK; ^2^ Cardiology Clinical Academic Group, Molecular and Clinical Sciences Research Institute St George's, University of London, St George's Hospital Grosvenor Wing Room 2.113, Blackshaw Road, Tooting London SW170QT UK; ^3^ Institute of Infection and Immunity St George's, University of London London UK

**Keywords:** Chronic kidney disease, Kidney failure, Heart failure, Angiotensin converting enzyme inhibitors, Aldosterone antagonists, Systolic heart failure

## Abstract

**Aims:**

CKD‐HF patients suffer excess hospitalization and mortality, often under‐treated with life‐prolonging medications due to fear of worsening renal function and hyperkalaemia. Yet, role of inter‐disciplinary working in improving therapy is unknown, which this study aims to investigate.

**Methods and results:**

Clinical, biochemical data, and medications at first and last clinic visit were obtained from patient records for 124 patients seen in kidney failure–heart failure clinic (23 March 2017 to 11 April 2019). Medication dose groups (none, low, and high dose), number of RAASi agents, and blood test results were compared between first and last visit in patients with at least two clinic visits (*n* = 97). Patient characteristics were age 78.5 years (IQR 68.1–84.4 years), male 67.7%, diabetes 51.6%, moderate (45.2%) vs. severe (39.5%) CKD, HF with reduced ejection fraction (HFrEF) (49.2%), follow‐up 234 days (IQR 121–441 days). HFrEF was associated with increased risk of death (adjusted OR 4.49, 95% CI 1.43–14.05; *P* = 0.01).

Distributions of patients according to number of RAASi agents they were on differed between first and last visit (*P* = 0.03). Dosage was increased in 25.9% for beta‐blockers, 33.0% for ACEi/ARBs, and 17.5% for MRAs. Distributions of patients across MRA dosage groups was different (*P* = 0.03), with higher proportions on higher dosages at last visit, without significant changes in serum potassium or creatinine. Serum ferritin improved (131.0 vs. 267.5 μg/L; *P* < 0.001), and fewer patients had iron deficiency (56.7% vs. 26.8%; *P* = 0.002) at last visit compared to the first.

**Conclusions:**

This inter‐disciplinary clinic improved guideline‐recommended medication prescription, MRA dosages in CKD‐HF patients without significant biochemical abnormality, and iron status. A prospectively designed study with medication titration protocol and defined patient‐centred outcomes is needed to further assess effectiveness of such clinic.

## Background

A population of patients with concurrent chronic kidney disease (CKD) and heart failure (HF) has been increasing due to each disease's increasing prevalence in the aging population as well as complex interactions between these two disease entities. Despite their well‐known survival benefits in HF patients, there is no clear guidance on the use of beta‐blockers, angiotensin‐converting enzyme inhibitors (ACEis), angiotensin‐receptor blockers (ARBs) and mineralo‐corticoid receptor antagonists (MRAs) in CKD–HF patients due to exclusion of severe CKD patients from major clinical trials.^1^ Clinicians often hesitate to initiate or up‐titrate renin‐angiotensin‐aldosterone system inhibitors (RAASi) due to concerns regarding potential deterioration in renal function or hyperkalaemia. CKD–HF patients, who already suffer high hospitalization and mortality,^1^ are often under‐treated with these life‐prolonging medications due to these challenges.^2^

A multi‐disciplinary approach has been recommended for management of CKD–HF patients due to demonstrated improved patient outcomes.^3^, ^4^, ^5^, ^6^ To our knowledge, there has never been an inter‐disciplinary clinic with input from nephrologist and cardiologist for joint decision regarding medication optimization. This report describes experience of such novel combined kidney failure–heart failure (KFHF) clinic, evaluating its effectiveness and exploring patient outcomes.

## Methods

Criteria for referral to kidney failure–heart failure (KFHF) clinic were concomitant CKD (stage 3 or above) and heart failure. Patients were followed up at varying frequencies as per clinical needs and discharged when they are stable on maximally tolerated therapy of life‐prolonging medications.

Patient demographics, clinical, and biochemical data were acquired from electronic records and clinic letters. Blood test results and doses of medications (beta‐blockers, ACEi/ARBs, and MRAs) were recorded for first and last clinic visits. Patients were categorized into three HF subgroups according to ejection fraction (EF): reduced (HFrEF) (EF < 40), mid‐range (HFmEF) (40 ≤ EF < 50) and preserved (HFpEF) (EF ≥ 50), and two CKD subgroups: moderate (stage 3) and severe (stage 4/5/dialysis).

Effect of different variables on death was analysed using univariate and multiple variable logistic regression analysis and presented by the resulting odds ratios (OR) and 95% confidence interval (95% CI).

Daily doses of individual therapeutic agents were categorized into none, low, and high dose (≥50% of maximum dose). Patients were categorized into none, single, and dual therapy depending on the number of RAASi agents they were on. These categorizations generated ordinal variables and were analysed accordingly.

## Results

KFHF clinic received 154 referrals from March 2017 to April 2019: 30 patients were excluded for non‐attendance or inappropriate referral; 124 patients were seen and followed up, of whom, 97 had had at least two clinic visits (hence, included in the analysis of medication titration and blood test results).

Patient characteristics were median age 78.5 years (IQR 68.1–84.4 years), male 67.7%, diabetes mellitus 51.6%, median follow‐up time 234 days (IQR 121–441 days, minimum 6 days, maximum 749 days). There was no difference among CKD or HF subgroups with regard to patient characteristics and baseline blood tests (*Table* *1*).

**TABLE 1:** Baseline characteristics of patients (*n* = 124) according to CKD and HF cohorts

	Overall	Moderate CKD	Severe CKD	*P* value	HFrEF	HFmEF	HFpEF	*P* value
Proportion	100% (124)	54.8% (68)	45.2% (56)	—	49.2% (61)	20.2% (25)	29.8% (37)	—
Age (years)	78.5 (16.3)	78.8 (13.7)	78.3 (19.9)	0.53	77.8 (17.6)	79.0 (61.2–83.4)	80.5 (14.8)	0.45
eGFR (mL/min/1.73m^2^)	31.4 (10.1)	38.4 (6.8)	22.1 (4.7)	<0.001	31.2 (10.3)	30.3 (11.1)	32.1 (9.1)	0.85
EF (%)	38.8 (13.8)	40.1 (14.9)	37.2 (12.3)	0.32	27.1 (6.6)	42.1 (2.5)	55.8 (5.2)	<0.001
Diabetes	52.4% (65)	52.9% (36)	51.8% (29)	0.90	44.3% (27)	64.0% (16)	59.5% (22)	0.20
Na (mmol/L)	140.5 (3.5)	140.2 (3.9)	140.8 (3.0)	0.61	140.3 (3.9)	141.6 (2.4)	140.1 (3.3)	0.26
K (mmol/L)	4.6 (0.5)	4.6 (0.5)	4.6 (0.6)	0.60	4.6 (0.5)	4.5 (0.5)	4.6 (0.6)	0.39
Hb (g/L)	114.1 (20.7)	117.3 (20.3)	110.3 (22.8)	0.07	114.9 (21.3)	113.4 (23.3)	113.0 (18.4)	0.66
Hospital admissions	50% (62)	42.6% (29)	58.9% (33)	0.10	60.7% (37)	32.0% (8)	45.9% (17)	0.04^*^
Death	16.1% (20)	14.7% (10)	17.9% (10)	0.81	24.6% (15)	8.0% (2)	8.1% (3)	0.04^*^

The continuous data are summarized as mean/SD if normally distributed or median/IQR interval otherwise. *P* values are the results of appropriate tests applied according to data nature, that is, *t*‐tests for continuous normally distributed data, Kruskal–Wallis for continuous variables which display departures from normality, and *χ* ^2^ for proportions.

^*^ *P* values were 0.03 and 0.02, respectively, for comparison of proportions of patients having hospital admission and death between HFrEF patients and those with non‐reduced EF.

### Inpatient admission and death

Patients with HFrEF were significantly more likely to have inpatient admissions compared to those with non‐reduced EF (60.7% vs. 40.3%; *P* = 0.03). They were also 4.5 times (95% CI 1.43–14.05; *P* = 0.01) more likely to die; this was adjusted for age, sex, diabetes, and CKD status (*Table* 2).

**TABLE 2:** Logistic regression analysis on effects of different variables on mortality

	Univariate analyses				Multiple variables analysis			
Variable	OR	*P* value	95% CI for OR		OR	*P* value	95% CI for OR	
Age (1 year)	1.04	0.11	0.99	1.09	1.05	0.07	1.00	1.10
Female	1.50	0.42	0.56	4.02	1.84	0.26	0.64	5.31
Diabetes	0.70	0.47	0.27	1.83	0.98	0.97	0.35	2.73
Severe CKD	1.26	0.64	0.48	3.29	1.19	0.74	0.43	3.27
HFrEF	3.72	0.02	1.26	10.99	4.49	0.01	1.43	14.05
Constant^a^					0.06	<0.001	0.17	0.23

^a^ Constant: odds of death for a male patient of median age (78.5 years), non‐diabetic, with moderate CKD and HF with ejection fraction >40.

There was no difference in the likelihood of having hospital admissions (42.6% vs. 58.9%; *P* = 0.10) or risk of death (17.9% vs. 14.7%; *P* = 0.81) between two CKD subgroups (*Table* 1).

### Medications

**FIGURE 1:**
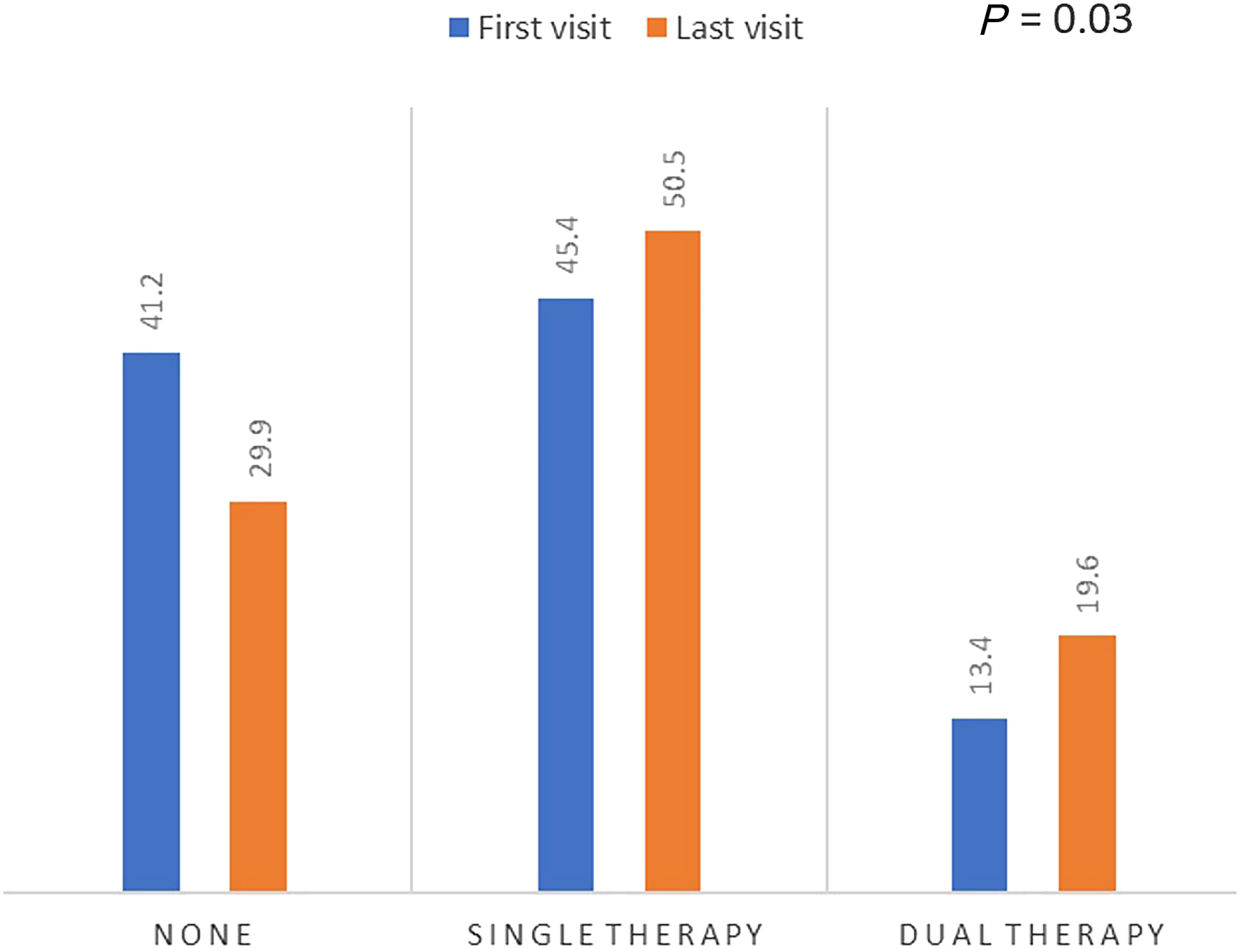
Comparison of proportions of patients according to number of renin‐angiotensin‐aldosterone‐system inhibitors (RAASi) agents used between the first and last visit (*P* = 0.03)

There was some evidence which supported a difference between the first and last visit with regard to number of RAASi agents used (*P* = 0.03). Proportions of patients on no RAASi decreased from 41.2% to 29.9% while those on single or dual therapy increased from 45.4% to 50.5% and 13.4% to 19.6%, respectively (*Figure* *1*). At the end of follow‐up, 7.2% of patients were receiving no key therapies (beta‐blockers or RAASi agents)**.**

**FIGURE 2:**
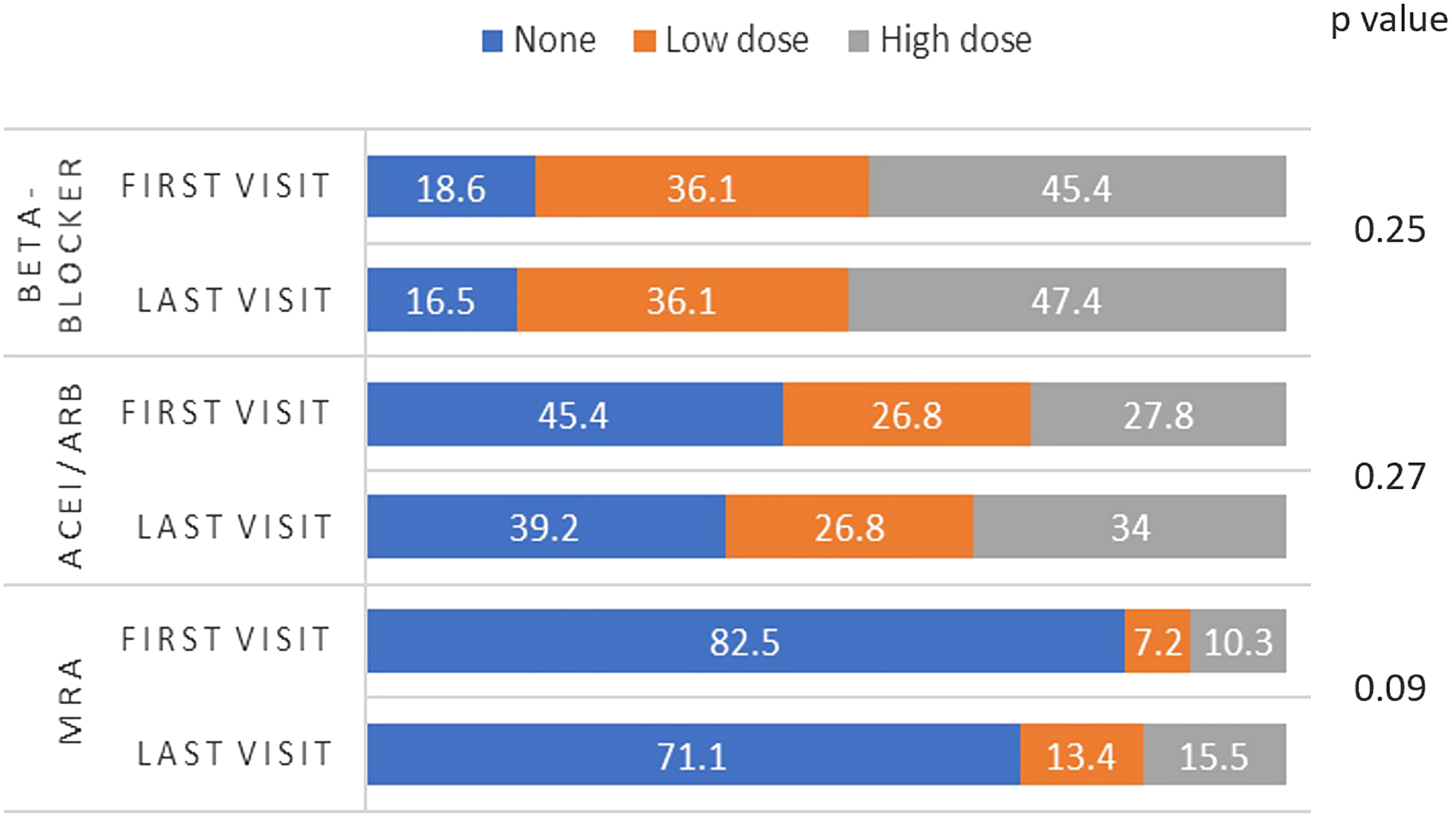
Comparison of proportions of patients in different medication dosage groups (none, low dose, and high dose) between the first and last visit

Dosage was increased in 25.9% of patients for beta‐blockers, 33.0% for ACEi/ARBs, and 17.5% for MRAs. There was no evidence to suggest any difference in distributions of patients across dosage groups for beta‐blockers (*P* = 0.46) and ACEi/ARBs (*P* = 0.20) (*Figure* *2*). The distribution of patients across MRA dose categories was different (*P* = 0.03), with more patients being on low and high dose at the last visit compared to the first (*Figure* 2). There was no significant difference in the likelihood of each medication dose being up‐titrated, across CKD and HF subgroups (*Table* *3*).

**TABLE 3:** Comparison of proportions of patients with successful medication dose up‐titration among CKD and HF subgroups

	Moderate CKD (%)	Severe CKD (%)	*P* value	HFrEF (%)	HFmEF(%)	HFpEF (%)	*P* value
Beta‐blockers	34.1	16.2	0.07	25.6	29.4	24.0	0.92
ACEi/ARBs	35.3	35.9	0.95	32.6	52.4	26.1	0.16
MRAs	18.2	16.7	0.85	19.1	23.8	10.3	0.43

### Electrolytes and renal function

There was no overall difference in serum potassium and creatinine level in patients whose ACEi/ARB and MRA dose was increased (*Table* *4*). Hyperkalaemia (≥5.5 mmol/L) was present in 6.2% of patients at baseline and 10.3% at the last visit (*P* = 0.29). Risk of hyperkalaemia was 6.1% among patients with ACEi/ARB dose up‐titrated, 6.3% in those with MRA dose up‐titrated.

**TABLE 4:** Comparison of renal function, serum sodium, potassium between the first and last clinic visit in all patients with at least two clinic visits (*n* = 97) or subgroups when specified

	First visit	Last visit	*P* value
Na (mmol/L)	140.5 (3.5)	139.6 (4.1)	0.01
K (mmol/L)	4.6 (0.5)	4.7 (0.6)	0.43
K (ACEi/ARBs increased)	4.6 (0.5)	4.7 (0.6)	0.71
K (MRAs increased)	4.5 (0.5)	4.7 (0.6)	0.25
Creatinine (μmol/L)	188.8 (64.4)	201.5 (81.1)	0.03
Creatinine (ACEi/ARBs increased)	185.1 (59.6)	192.0 (68.9)	0.17
Creatinine (MRAs increased)	163.5 (47.8)	175.5 (36.5)	0.97
eGFR (mL/min/1.73m^2^)	31.5 (10.1)	29.8 (11.2)	0.02
eGFR (ACEi/ARBs increased)	32.5 (10.5)	31.6 (11.3)	0.23
eGFR (MRAs increased)	36.3 (9.4)	32.1 (6.9)	0.02
CKD stages			
Stage 3	56.7% (55)	48.5% (47)	
Stage 4	38.1% (37)	37.1% (36)	
Stage 5	2.1% (2)	8.2% (8)	
Dialysis	3.1% (3)	6.2% (6)	
Hb (g/L)	114.8 (20.6)	116.3 (19.7)	0.84
Anaemia (Hb <100 g/L)	17.5%	20.6%	0.65
Hb (anaemic patients at first visit)	85.9 (12.8)	100.8 (18.1)	0.02
Ferritin (μg/L)	131.0 (220.0)	267.5 (359.0)	<0.001
Iron deficiency	56.7% (55)	26.8% (26)	0.002
Ferritin (iron deficiency group at first visit)	67.0 (62.0)	185.0 (269.0)	<0.001

Results are displayed as mean (SD), median (IQR interval), or percentages.

### Haemoglobin and iron management

In patients who were anaemic at the first clinic visit, mean serum haemoglobin level increased (85.9 to 100.8 g/L; *P* = 0.02). EPO was given to two patients.

Overall serum ferritin level increased between the first and last clinic (131.0 vs. 267.5 μg/L; *P* ≤ 0.001) and proportion of patients with iron deficiency decreased from 56.7% to 26.8% (*P* = 0.002). Of those with iron deficiency at baseline, 43.6% received IV iron at the same clinic visit, with a significant increase in ferritin level (67.0 to 185.0 μg/L; *P* < 0.001).

## Discussion

This study reports outcomes from the first 2 years of a novel combined kidney failure and heart failure clinic and its attempt to improve prescription and up‐titrate dosage of life‐prolonging medications in a real‐world cohort of patients with concomitant moderate or severe CKD and HF. More patients were on single or dual RAASi therapy at their last visit comparing to the first; the difference in distributions was statistically different. The distribution of patients according to dosage groups was different between the first and last visit for MRAs, with higher proportions of patients being on higher dosages while having no associated clinically significant deterioration in renal function and hyperkalaemia.

There are very few studies looking at medication prescription in a similar outpatient CKD–HF cohort which we can compare our results with. In a study by Frohlich *et al*. looking retrospectively at ACEi/ARB usage in an outpatient HF clinic, ACEi/ARB dose was successfully increased in 37.3% of eligible patients^7^ which is higher than our rate of 33.0%. This difference could be explained by their exclusion of CKD stage 5 and dialysis patients and the fact that their follow‐up period was fixed at 12 months.^7^ The varying follow‐up period in our study meant that a proportion of patients was at early stages of medication optimization. With regard to other possible factors affecting medication optimization, our study showed that severity of CKD and nature of HF did not have an influence on the likelihood of successful initiation or dose up‐titration similar to a study by Heywood *et al*.^2^

Risk of hyperkalaemia in patients with successful RAASi dose up‐titration in our study was comparable to that reported in clinical trials. Reported risk of RAASi‐related hyperkalaemia in clinical trials varies depending on study settings, baseline renal function, and severity of HF and can range from 3% to 7% for ACEi/ARBs^8^, ^9^, ^10^, ^11^ and 2% to 8.0% for MRAs.^12^, ^13^, ^14^ Trials with lower rates of hyperkalaemia have stricter definition for hyperkalaemia (>6 mmol/L) such as Candesartan in Heart failure Assessment of mortality and Morbidity (CHARM) and Randomized Aldactone Evaluation Study (RALES)^10^, ^11^, ^13^ or lower proportion of patients with CKD (33–48%)^1^ compared to our patient group (100%).

As the analysis looks at only two time points, this study is unable to capture the complexity of patient management and medication trials through the length of follow‐up. As the result, worsening renal failure or hyperkalaemia happening in between these two end points can still be reason behind failure of initiation or dose titration.

There is currently limited research into prognosis of CKD–HF patients according to HF groups. Two of such studies have reported different outcomes: a study by Lofman *et al*. found similar 1 year mortality rates in all HF groups^15^ while in Ahmed *et al*., CKD patients with systolic HF seem to do worse than those with diastolic HF.^16^ Patients with HFrEF in our study suffered the worst outcomes with significantly higher rates of hospital admissions and death. To further assess the effectiveness of this novel clinic, it would be beneficial to conduct a prospective study to compare outcomes of our patient population to a matched cohort being followed up in general nephrology and heart failure clinic.

The fact that patients attending this clinic benefit from anaemia nurse specialist input and same‐day intravenous Iron administration also means more efficient use of healthcare resource, minimization of patients' waiting time, transport time, and expense.

## Conclusions

This is an initial report on a novel inter‐disciplinary kidney failure and heart failure clinic, which had improved prescription of RAASi agents and MRA dosages in a cohort of patients with CKD and HF, without resulting clinically significant biochemical abnormalities. The effectiveness of such clinic can be further assessed using a prospective study monitoring medication titration steps, related adverse events, patients’ satisfaction, as well as outcomes including quality of life, hospitalization, and mortality rate.

## Conflict of interest

DB has received Grants from British Heart Foundation PG 10/71/28462 and partially funded for this work by Welcome ISSF; DB has received Honoraria from Pfizer, ViforPharma and AstraZeneca.

MN was partially funded by George's Academic Training (GAT).

## Supporting information


**Data S1.** Supporting Information
