# Supplementary material for: Management and outcomes of heart failure patients with CKD: experience from an inter‐disciplinary clinic
Source: ESC Heart Fail. 2020 Jul 11;7(5):3225–30. doi: 10.1002/ehf2.12796 (PMC7524254; doi:10.1002/ehf2.12796)
Supplement: Supplementary file 1 — Data S1. Supporting Information [file EHF2-7-3225-s001.docx]

Supplementary file ESCHF-20-00158: **Statistics**

**Proportions of patients with medication dose increased**

|  | **HFrEF** | **HFmEF** | **HFrEF + HFmEF** | **HFpEF** |
| --- | --- | --- | --- | --- |
| **Beta-blockers** | 25.6% | 29.4% | 39.4% | 24.0% |
| **ACEi/ARBs** | 32.6% | 52.4% | 38.8% | 26.1% |
| **MRAs** | 19.1% | 23.8% | 20.6% | 10.3% |

**Analysis include only patients with HFrEF: n = 47**

No difference in number of RAASi agents, number of key therapies (including beta-blockers) patients were on between first and last visit.
No difference in distribution of dose groups between first and last visit.

| **Number of RAASi agents** | **0** | **1** | **2** | **p value** |
| --- | --- | --- | --- | --- |
| First visit | 46.8% | 31.9% | 21.3% | 0.11 |
| Last visit | 36.2% | 34.0% | 29.8% |  |

| **Number of key therapies** | **0** | **1** | **2** | **3** | **p value** |
| --- | --- | --- | --- | --- | --- |
| First visit | 10.6% | 36.2% | 31.9% | 21.3% | 0.09 |
| Last visit | 4.3% | 36.2% | 29.8% | 29.8% |  |

| **Medication dose group** | | **None** | **Low dose** | **High dose** | **p value** |
| --- | --- | --- | --- | --- | --- |
| Beta-blockers | First visit | 10.6% | 51.1% | 38.3% | 0.32 |
|  | Last visit | 8.5% | 48.9% | 42.6% |  |
| ACEi/ARBs | First visit | 51.1% | 25.5% | 23.4% | 0.20 |
|  | Last visit | 42.6% | 27.7% | 29.8% |  |
| MRAs | First visit | 74.5% | 8.5% | 17.0% | 0.21 |
|  | Last visit | 63.8% | 17.0% | 19.1% |  |

**Analysis include only patients with HFrEF and HFmEF: n = 68**

Difference in number of key therapies (including beta-blockers) patients were on (p=0.04)

No difference in distribution of dose groups or number of RAASi agents patients were on between first and last visit

| **Number of RAASi agents** | **0** | **1** | **2** | **p value** |
| --- | --- | --- | --- | --- |
| First visit | 42.6% | 39.7% | 17.6% | 0.06 |
| Last visit | 30.9% | 44.1% | 25.0% |  |

| **Number of key therapies** | **0** | **1** | **2** | **3** | **p value** |
| --- | --- | --- | --- | --- | --- |
| First visit | 8.8% | 36.8% | 38.2% | 16.2% | 0.04 |
| Last visit | 5.9% | 29.4% | 41.2% | 23.5% |  |

| **Medication dose group** | | **None** | **Low dose** | **High dose** | **p value** |
| --- | --- | --- | --- | --- | --- |
| Beta-blockers | First visit | 13.2% | 42.6% | 44.1% | 0.44 |
|  | Last visit | 11.8% | 41.2% | 47.1% |  |
| ACEi/ARBs | First visit | 47.1% | 26.5% | 26.5% | 0.27 |
|  | Last visit | 39.7% | 27.9% | 32.4% |  |
| MRAs | First visit | 77.9% | 8.8% | 13.2% | 0.09 |
|  | Last visit | 66.2% | 16.2% | 17.6% |  |

Supplementary file ESCHF-20-00158:

**Protocol for management of CKD Heart Failure patients**

**Maximisation of ACE-inhibitors, Angiotensin Receptor Blockers and Mineralocorticoid Receptor Antagonists**

Maximise the ACE-inhibitors (ACEi), Angiotensin Receptor Blockers (ARB) and Mineralocorticoid Receptor Antagonists (MRA) in patients with reduced ejection fraction with careful monitoring of blood potassium and kidney function.

Check serum potassium and creatinine 2 weeks after initiation and escalation of ACEi/ARB or MRA.

Allow a rise in serum creatinine upto 30%.

Allow the rise of potassium to 5.6 mmol/L.

Discontinue or decrease dose of ACEi/ARB or MRA if serum potassium >5.9 mmol/L.

Advise low potassium diet if serum potassium is >5.6 mmol/L.

Correct acidosis with Sodium Bicarbonate if serum bicarbonate <21 mmol/L.

**Intravenous iron**

Investigate for alternative causes of anaemia such as blood loss, vitamin B12 deficiency, folate deficiency and haemolysis.

Consider use of intravenous iron if serum ferritin <100 mg/L or TSAT<20% and haemoglobin <140g/L.

Consider starting EPO if Haemoglobin<100g/L, eGFR <45 ml/min/1.73m^2^ and iron stores are replenished.

**Diuretics**

Use diuretics for lower limb oedema and pulmonary oedema.

Use loop diuretics rather than thiazide as first line with eGFR <30 ml/min/1.73m^2^

Consider using combination therapy with potassium sparing diuretic.

Consider using metalazone with loop diuretic for resistant oedema with careful monitoring of serum sodium, potassium and creatinine.
